# Switched CMOS current source compared to enhanced Howland circuit for bio-impedance applications

**DOI:** 10.2478/joeb-2024-0017

**Published:** 2024-10-05

**Authors:** Pablo Dutra da Silva, Pedro Bertemes Filho

**Affiliations:** 1 Electrical Engineering Department, State University of Santa Catarina, Mexico, Brazil

**Keywords:** Bio-impedance Spectroscopy, CMOS Integrated Circuit, Analog Front-end, Current Source

## Abstract

Bio-impedance Spectroscopy (BIS) is a technique that allows tissue analysis to diagnose a variety of diseases, such as medical imaging, cancer diagnosis, muscle fatigue detection, glucose measurement, and others under research. The development of CMOS integrated circuit front-ends for bioimpedance analysis is required by the increasing use of wearable devices in the healthcare field, as they offer key features for battery-powered wearable devices. These features include high miniaturization, low power consumption, and low voltage power supply. A key circuit in BIS systems is the current source, and one of the most common topology is the Enhanced Howland Current Source (EHCS). EHCS is also used when the current driver is driven by a pseudo-random signal like discrete interval binary sequences (DIBS), which, due to its broadband nature, requires high performance operational amplifiers. These facts lead to the need for a current source more compatible with DIBS signals, ultra-low power supply, standard CMOS integrated circuit, output current amplitude independent of input voltage amplitude, high output impedance, high load capability, high output voltage swing, and the possibility of tetra-polar BIS analysis, that is a pseudotetra-polar in the case of EHCS. The objective of this work is to evaluate the performance of the Switching CMOS Current Source (SCMOSCS) over EHCS using a Cole-skin model as a load using SPICE simulations (DC and AC sweeps and transient analysis). The SCMOSCS demonstrated an output impedance of more than 20 *M*Ω, a ± 2.5 *V* output voltage swing from a +3.3 V supply, a 275 *μA* current consumption, and a 10 *k*Ω load capacity. These results contrast with the + 1.5 V output voltage swing, the 3 *k*Ω load capacity, and the 4.9 *mA* current of the EHCS case.

## Introduction

Wearables are devices that can measure biological signals and activity patterns found as wristbands, smartwatches, chest straps, shoes, socks, and glasses. Originally designed to monitor activity patterns such as sleep, walking, or running, they are now capable of quantifying a variety of vital signs and are used in healthcare tracking, but not for medical diagnostics. However, recent scientific research has begun to explore the possibility of establishing correlations between activity patterns, which are linked to lifestyle, and certain cardiovascular diseases [1, 2, 3]. Wearable technology has the potential to provide non-invasive, real-time monitoring of patients in their homes or in an ambulatory setting, as well as early detection of health issues, and tracking performance enhancement for athletes [1].

The wearable system must have an ultra-low voltage supply, ultra-low power consumption, small volume, and high precision, as stated in references [1, 2, 3]. The convergence of these characteristics is found in very large-scale integration (VLSI) fabrication technology, such as complementary metal-oxide semiconductor (CMOS), which is a standard technology for fabricating integrated circuits (ICs). Various research works propose different integrated circuit designs for bio-impedance spectroscopy applications [4, 5]. In addition, some solutions on the market can be cited, such as Analog Devices’ MAX30009, as an indication of efforts to develop a bio-impedance analyser for wearable devices.

Impedance values are influenced by the frequency at which they are measured and are examined within a specific frequency range. The examination can be carried out either in the time domain or in the frequency domain. In the time domain approach, a pseudo-random binary signal (DIBS) is utilized, which possesses a power spectral density that is uniformly distributed across a given frequency band. This signal is then applied to the sample, and the resulting measurement is sampled and digitally processed to determine the relationship between impedance and frequency. On the other hand, frequency domain analysis involves applying a sine signal with incremental changes in frequency and recording the corresponding impedance values for each frequency applied [6, 7, 8].

In order to measure impedance, BIS commonly relies on a current source to inject current into a sample and then measures the resulting voltage. Linear circuits, such as operational amplifiers or transconductance amplifiers, are frequently employed to generate current sources. Even when utilizing pulsed signals, such as pseudorandom signals, linear sources are typically preferred for this purpose. Several studies have highlighted the performance and design aspects of current sources in BIS [9, 10, 7, 11, 12, 13, 14, 4, 15, 16, 17].

The objective of this study is to conduct a SPICE simulation to compare the performance of a switching CMOS current source (SCMOSCS), that is a switched circuit, and an EHCS. The SCMOSCS configuration is based on a high-impedance current mirror and an H-bridge that utilizes CMOS inverters, similar to the I-DAC system described in [5]. This proposed design is particularly suitable for integrated circuit design and offers several advantages over the utilization of linear circuits for time-domain BIS. However, the SCMOSCS currently lacks established design equations, which could potentially hinder optimization efforts. Consequently, the development of these equations will be the primary focus of future research endeavors.

This research examines an Enhanced Howland current source specifically created for low voltage power supplies operating within a 100 kHz to 1 MHz DIBS signal band and compares with the SCMOSCS topology. In order to assess the effectiveness of the current sources and confirm the suitability of SCMOSCS for this particular application, various analyses including DC and AC sweeps, transient, and FFT analyses were conducted on the current, voltage, and impedance measurements of a Cole [18] electric model arranged in a tetra-polar BIS arrangement.

## Topologies under comparison

**Figure 1:**
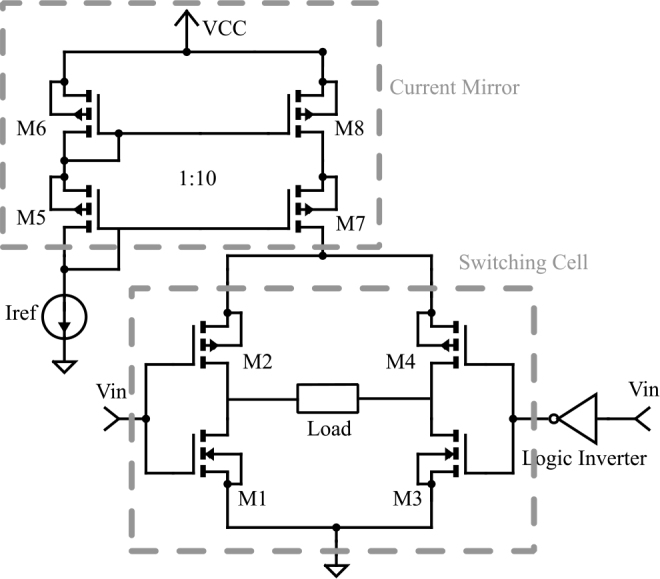
SCMOSCS Schematic Diagram.

The circuit illustrated in Fig. 1 utilizes a PMOS cascode current mirror, which is powered by the VCC voltage, along with a DC programmed current source (Iref). This combination ensures a constant and programmed output current that remains unaffected by the Discrete Interval Binary Sequences (DIBS) signal voltage [7, 12].

The H-bridge, consisting of CMOS inverters, is an essential component of this circuit. Its primary function is to change the mirror’s current direction through the load in response to variations in the DIBS signal levels in input. To operate the H-bridge effectively, a complementary switching signal is necessary. Hence, a CMOS inverter is employed on the right side of the bridge. When the DIBS signal is high, the complementary DIBS signal is low. As a result, transistors M1 and M4 are turned on, allowing the mirror’s current to flow from right to left through the load. Conversely, when the signal changes state, transistors M2 and M3 take over the current flow through the load, directing it from left to right. The output voltage is the differential voltage measured in load’s terminals. The accuracy of the output current, which is set by Iref, depends on the load impedance and the output impedance of the SCMOSCS, as it does for the EHCS. In addition, unlike the EHCS where the output current depends on the level of the input voltage signal, the output current accuracy of the SCMOSCS is independent of the amplitude, rise time and fall time of the input voltage source (DIBS signal).

The cascode mirror depicted in Fig. 1 was implemented to effectively increase the output impedance of the current source. The reference current (Iref) was adjusted to 25 *μA*, a value significantly lower than the 250 *μA* current that is the same amplitude set in EHCS case. This current gain provided by the mirror, as depicted in Fig. 1, plays a crucial role in reducing the power consumption of the device, making it suitable for battery-operated applications. The output current amplitude of 250 *μA* was set for both EHCS and SCMOSCS to facilitate a fair performance evaluation under identical conditions. The sizing of the mirror transistors was determined using the ACM transistor model [19] and technology parameters (180 *nm*) to ensure operation in the moderate inversion region (*i_f_* = 33), where the saturation voltage is approximately 150 mV as calculated by the equation (1) where *ϕ_t_* is the thermal potential. The inversion level of a saturated MOSFET is the relation *i_f_* = *I_D_*/(*I_sq_*.(*W/L*)) where *I_SQ_* is a technological parameter called specific current, *I_D_* is the drain current, and (*W/L*) is the aspect ratio of the transistors. The drain current and the specific current are used to determine the aspect ratio of transistors M5 to M7 shown in Table 1.

1 $$V_{DSsat}=\phi_{t\cdot }\left(\sqrt{1+i_{f}}+3\right)$$

**Table 1:** Transistor dimensions used in simulation

MOSFET	L (*μ*m)	W (*μ*m)
M1 and M3	1	40
M2 and M4	1	120
M5 and M6	2	48
M7 and M8	2	480

This selection criterion enables a wider voltage range in the load (tissue sample for analysis). Special attention was paid to the switching speed and on-state resistance of each transistor in the H-bridge configuration, M1 to M4 in Fig. 1, and one can observe in (2), where $\mu .C_{ox}^{\prime }$ is the product of the charge mobility and oxide capacitance, the (*W/L*) is the transistor’s aspect ratio, *V*_*T*0_ is the threshold voltage and *n* is the slope factor. All these cited parameters are fabrication technology dependent and the only design dependent is the aspect ratio (*W/L*).

2 $$R_{DS}=\frac{1}{\mu .C_{ox}^{\prime }.\left(W\text{/}L\right).\left(V_{G}-V_{T0}-nV_{S}\right)}$$

When developing a DIBS signal logic inverter (see Fig. 1), it is essential to take into account the switching speed and signal propagation time that is related with the transistor area of the logic gate. To meet the desired speed requirements, a digital cell designed for the maximum speed (small possible area) of the technology can be utilized [19].

**Figure 2:**
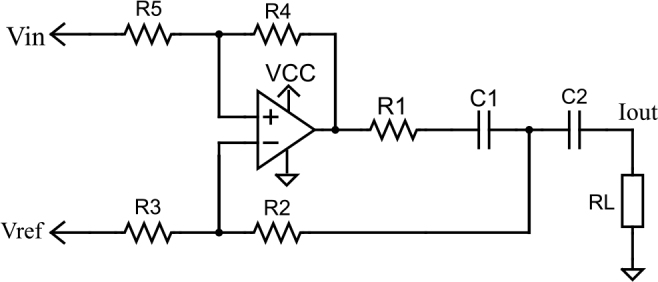
Enhanced Howland Current source Schematic.

The EHCS can be observed in Fig. 2. This configuration is frequently utilized in time or frequency domain impedance spectroscopy for injecting current into the sample [7, 5, 20, 21, 11]. When resistors are matched, the EHCS is recognized for its high output impedance. As a result, tolerances of less than 0.1% are necessary when employing discrete resistors [22, 23, 24]. The relationship between resistor tolerance and the output of the current source can be investigated by performing a Monte Carlo simulation. In PSPICE simulations, we can use the opamp macromodel supplied by the manufacturer, obtaining an output current variation between 246 to 267 *μA* for a tolerance of 1%, whereas 249.7 to 250.55 *μA* for 0.1%.

In both, frequency and time domain spectrographic analysis, the EHCS is appropriate for generating sinusoidal and pseudo-random current signals. It is crucial to carefully assess the gain-bandwidth product and slew rate of the operational amplifier when utilized in time domain BIS. The equations governing the design of the EHCS are detailed in the literature concerning impedance spectroscopy and will not be discussed here. The output current, see equation (3), is determined by the relationship between the input differential voltage and resistor R1 when *R*4 = *R*5 and *R*3 = *R*2 + *R*1 as illustrated in Fig. 2 [22, 23, 24]. Another aspect is that the input voltage source must be very accurate due to the relationship of the EHCS as a voltage-to-current converter.

3 $$I\;out=\frac{Vin-Vref}{R1}$$

In both cases, the system should fulfil the following specifications: single power supply of +3.3 *V*; output current of approximately 250 *μAp*; an output impedance higher than 10*M*Ω; single input pulsed signal (DIBS) with a maximum amplitude of +3.3 *V*.

The operational amplifier OPA2354 features a gainbandwidth product of 250 *MHz* and a slew rate of 150 *V/μs*. It can function in a rail-to-rail configuration at the output and necessitates a minimum single supply voltage of 2.5 *V*. These specifications are ideal for effectively processing DIBS signals with frequencies of up to 1 *MHz*. Notably, this amplifier consumes a quiescent current of 4.9 mA, which is approximately twenty times the specified output current and it is consumed all the time the circuit is on. The design of the EHCS source’s output current considers the voltage levels of the input signal (*Vin_high_* = VCC and *Vref* = VCC/2) and their relationship to resistor R1, following the equation (3). This calculation yields a resistor value of 6.6 *k*Ω. The design is finalized with resistors *R*4 = *R*5 = 10 *k*Ω, *R*2 = 100 Ω, and *R*3 = 6.7 *k*Ω, as well as the incorporation of DC decoupling capacitors *C*1 = *C*2 = 20 *μF*.

**Figure 3:**
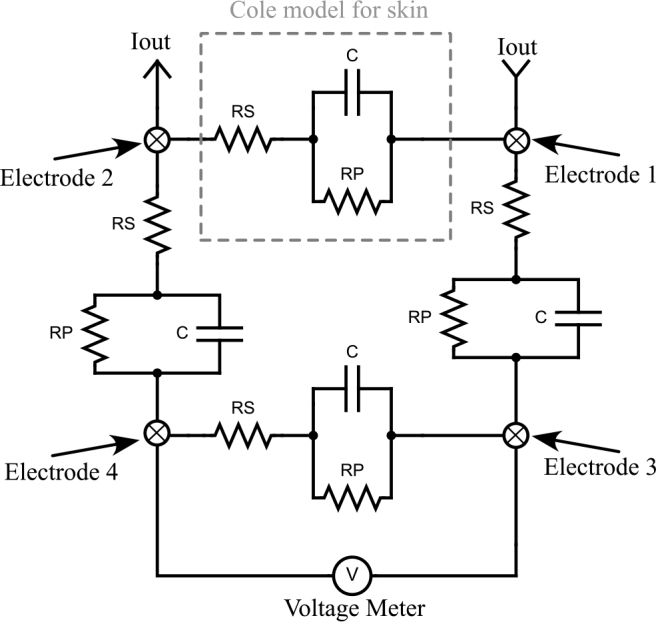
Load arrangement using cole model.

The tetra-polar arrangement of the electrode for BIS is shown in Fig. 3 and the Cole electrical model for human tissue can be seen in the highlight. In addition, three replicas have been distributed between the electrodes. These replicas take into account an isometric electrical behaviour of the tissue. Electric current is injected into electrode 1 and leaves the load through electrode 2. The voltage is measured between electrodes 3 and 4. The resistors *RS* = *RP* = 1 *k*Ω and a capacitor of 1 *nF* are also used in this work.

## Results and Discussions

**Figure 4:**
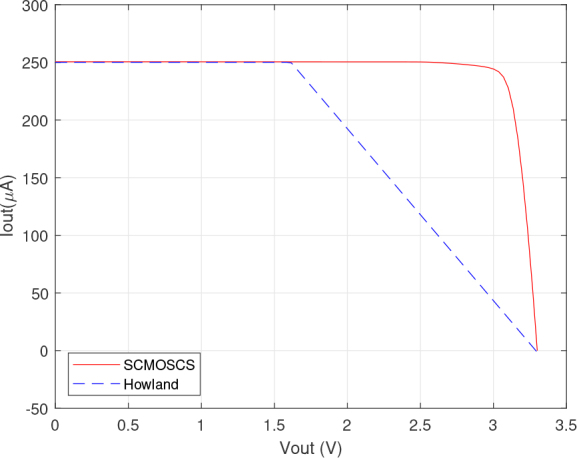
DC analysis results of the Output current versus output voltage.

**Figure 5:**
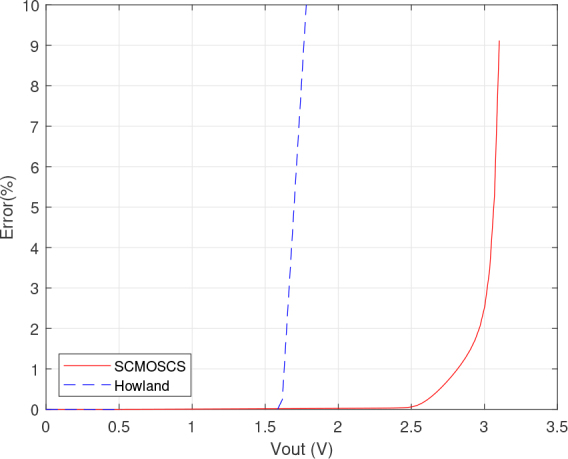
DC analysis results of the output current relative error versus output voltage.

**Figure 6:**
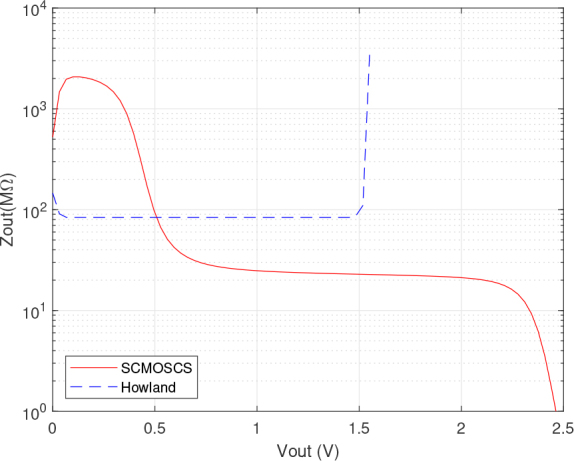
DC analysis results of the output impedance versus output voltage.

**Figure 7:**
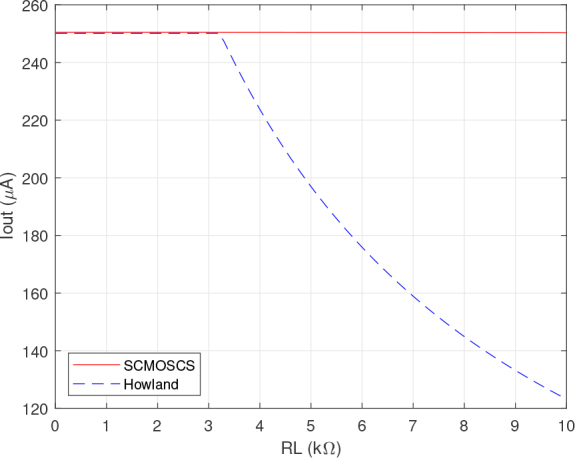
DC analysis results of the output current versus load resistance

The results depicted in Fig. 4, Fig. 5, Fig. 6 and Fig. 7 were obtained by Cadence PSPICE simulation using the opamp macromodel supplied by the manufacturer for the EHCS case. The SCMOSCS was simulated in Cadence Spectre using the UMC 180 *nm* process design kit. The correlation between the output DC current and output DC voltage sweep is obtained when a voltage source is connected to the load electrodes and its results are illustrated in Fig. 4, Fig. 5, and 6. The experiment showcases the permissible output voltage swing for both scenarios of current sources. The programmed output current had a relative error to the specification of less than 0.1 % within the output voltage swing in both current sources. Fig. 4 and Fig. 5 distinctly highlights the superiority of the SCMOSCS, which boasts a higher output voltage swing of ± 2.5 *V* due to the minimal potential to saturate the cascode mirror given by $V_{O_{min}}=V_{G_{M6}}+2.V_{DS_{sat}}$. The SCMOSCS features a swing of ±2.5 V, approximately 3.33 times greater than the EHCS swing of +1.6 *V*. It is important to mention that the outcomes presented in Fig. 4, Fig. 5 and Fig. 6 are symmetrical in relation to the y-axis only for the SCMOSCS case.

Upon analysing Fig. 6, which displays the DC relationship between output impedance and output voltage, it is evident that the output impedance of SCMOSCS reaches approximately 2 *G*Ω for *Vout* values less than 300 mV. Probably, it is related to a small region where the H-bridge PMOS transistors are in saturation due to the low voltage in the load terminals. Above the mentioned output voltage, the output impedance is dominated by the mirror, which has a value of more than 20 M*Ω* and presents a slightly reduction until the output voltage reaches 2.2 *V*. This reduction is explained by the dependence of the early voltage due to channel length modulation second order effect. As the maximum load impedance is much lower than the output impedance, this behaviour does not affect SCMOSCS performance. This value match with the theoretical CC output impedance for this cascode mirror given by *R_out_* = *g_ms_/g_md_*^2^ [19]. The transconductances *g_ms_* and *g_md_* can be designed with the transistor’s dimensions and technological second order parameters as its early voltage [25].

The saturation effect seen in Fig. 6 does not affect the accuracy of the source as can be seen in Fig. 4 and Fig. 5. Although the EHCS has a higher output impedance in a certain DC operating range, this is dependent on the tolerance of the resistor as well as the second-order effects of the opamp. The SCMOSCS has the advantage that the high impedance can be defined by the design and not by resistor tolerance, resulting in a higher output voltage swing than the EHCS. This higher output voltage swing allows a higher load capacity for the SCMOCSC source compared to the EHCS, as shown in Fig. 7 in the DC RL sweep. All of these DC sweep tests were performed with the circuits set up to provide a DC output current and with a resistive load or voltage source connected as a load.

To assess the sensitivity of the topologies to temperature drift and voltage supply fluctuations, these minimalistic corners simulations are conducted. Table 2 presents the verification of the DC output current results for the specified temperature range of 0 to 100°C and voltage supply range of 1.8 to 3.3 *V*. It can be observed that there is no significant variation in the temperature and supply voltage range analyzed.

**Table 2:** CC output current (in *μ*A) considering temperature and voltage supply corners

Topology	lout (0 < *T* < 100°*C*)	lout (1.8 < VCC < 3.3 *V*)
EHCS	250.118 to 250.121	249.97 to 250.12
SCMOSCS	250.35 to 250.22	247.95 to 250.33

Monte Carlo simulations were carried out to understand the effect of component variability on both the EHCS and the SCMOSCS. In the case of the EHCS, this simulation was already mentioned in the previous section and for the 0.1% tolerance resistors a variation from 249.7 *μA* to 250.55 *μA* for the value of the output current levels. For the SCMOSCS, a Monte Carlo simulation of 1000 iterations was performed, taking into account process and transistor mismatch variations. In this Monte Carlo analysis, a transient simulation was performed where the maximum variance in the output current level was 247.41 *μA* to 253.26*μA*. The difference in signal transition times was also evaluated with a range of approximately 5 *ns* between the shortest and longest transition time. These results show that the SCMOSCS is as robust as the EHCS in the corners analysed and in the Monte Carlo analysis.

Mismatch and process variations can’t significantly affect the accuracy of the current mirror in CMOS integration technology. This performance is due to the use of the same W/L transistors for both arms of the mirror. In this case, the gain is obtained by the number of transistors connected in parallel. The long length of the transistors, which increases their area, contributes to small mismatch deviations [19]. In addition, layout techniques used in the prototypes will maintain accuracy and minimise transistor mismatch [19].

Since the SCMOSCS current source has a good accuracy for reproducing the Iref current, the accuracy of the current source, with respect to the value of the designed output current level, depends on its reference. This should be independent of temperature, supply voltage and process variation. There are many current references in the literature with accuracy varying from 1 to 20% [26]. If the current reference does not achieve the required accuracy, the exactness of the system may be improved by measuring both the injecting current across the load and the load voltage. This arrangement is very common many in electrical bio-impedance spectroscopy systems [27].

**Figure 8:**
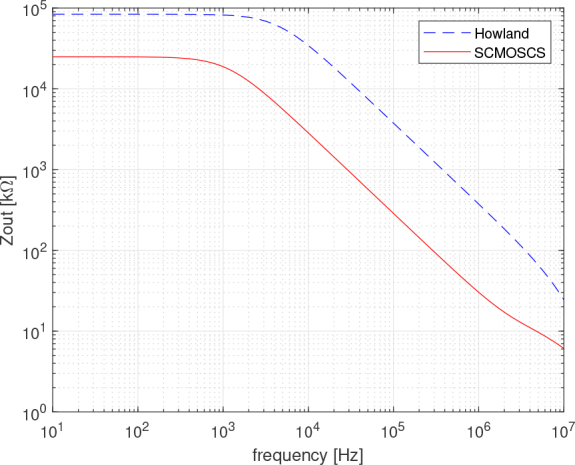
Output impedance versus frequency in AC analysis.

The AC analysis results for the output impedance are shown in Fig. 8. This was done using the same circuit setup as for the DC analysis, with the load changed by a signal generator in SPICE and an AC sweep analysis performed. The EHCS shows better performance from DC to high frequency, but the values are only relative to the op amp performance. It is important to note that the resistor tolerances affect the output impedance of the EHCS and degrade it. Due to the dominance of the mirror, the output impedance performance of the SCMOSCS can be designed and optimized by transistor sizing and modeled in simulation. In addition, a set of design equations could be a helpful tool to do this design optimization and it is in development.

**Figure 9:**
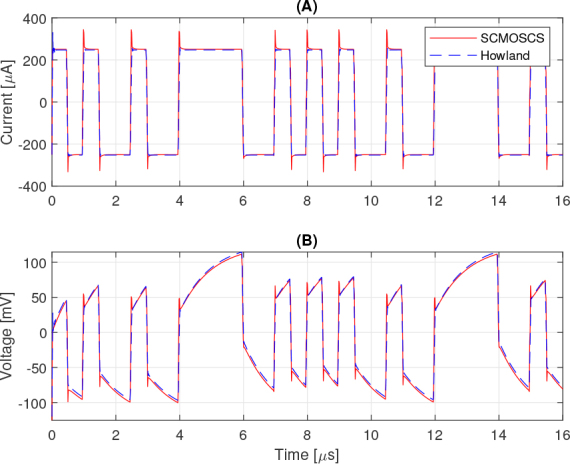
Transient analysis of: (A) output current and (B) output voltage.

Looking at the impedance differences between the two circuits in a frequency range from 0.1 to 1 *MHz*, the output impedance of the SCMOSCS is 30 *k*Ω at 1 *MHz*, whereas 300 *k*Ω for the EHCS. With this large difference, it was expected that there would be a comparative mismatch in transient analysis, that is shown in Fig. 9, but one can not verify any significant divergence between the curves. Another aspect to consider is the fact that a fair comparison of performance only consider the pseudo-random signals for both circuits, and not just the application of the sinusoidal signal, as the AC sweep analysis assumes.

The Fig. 9 shows the transient analysis for a 0.1 to 1 *MHz* band DIBS signal comprising 8 discrete frequencies separated by 125 *kHz*. This signal is pseudorandomly distributed over a time interval of 16 *μ*s and has a distribution of 32 levels, which can be high or low. Each level, during which the current remains constant at the programmed current, lasts 500 *μs*. For the tests carried out, the transition time between these levels was 50 *ns*. To obtain the impedance measured by electrical impedance spectroscopy, a test was carried out by connecting the electrodes 1 and 2, shown in Fig. 3, in the load terminals of both current source and the voltage have been measured in electrodes 3 and 4, presented in Fig. 3.

**Figure 10:**
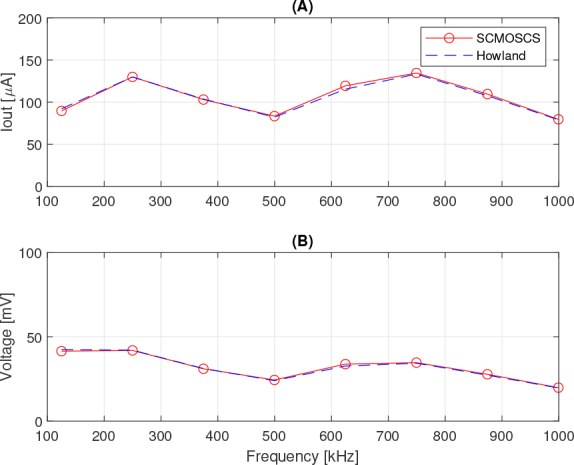
Spectra as function of frequency of: (A) output current and (B) output voltage.

**Figure 11:**
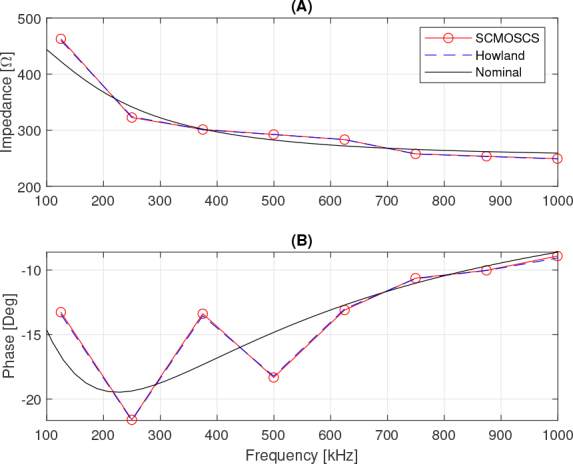
Impedance spectra as a function of frequency.

The current levels that can be verified in Fig. 9 (A) are ±250.49 *μA* for the SCMOSCS source and ±249 *μA* for the EHCS. These results show that for the type of signal with pulsed characteristics and for the analysis of a switching circuit, which is the case of the SCMOSCS source, the output impedance analysis in the frequency domain shown in Fig. 8 is not the most appropriate. There should be some difference between the responses within the band tested, because if we also consider the calculation of the error in the output current of a limited impedance current source, a load current of 238 *μA* can be calculated when the output impedance is 30 *k*Ω and the load is 1.5 *k*Ω, which is the equivalent impedance of the Cole model at low frequencies between electrodes one and two. The output current should be 243 *μA* taking into account the 750Ω equivalent impedance of the Cole model at high frequencies. Taking into account the theoretical value of the source of 250 *μA*, with an output impedance of 300 *k*Ω and a load of 1.5 *k*Ω, the current would be 248.75 *μA* in the case of EHCS. The equivalence between the two sources in terms of output impedance error can be seen in Fig. 9 (A) and Fig. 10 (A), where the spectrum is very close over the whole frequency band, despite the difference in output impedance in the AC analysis of Fig. 8. The spectrum (FFT) signal processing was done in Matlab and the DIBS’ frequencies are marked with a circle in Fig. 10 and Fig. 11. As mentioned above, one can not verify any significant difference between the circuits tested concluding that both circuits are equivalent to make the BIS analysis and the SCMOSCS presents advantages in power consumption (the OPA2354 uses 4.9 *mA* to bias, while the SCMOSCS uses 250 *μA*, 19.6 times more), VLSI design suitable and is suitable to higher frequency bandwidths.

The effect of the load’s capacitive parcel can be observed in Fig. 9 (B) and in its FFT in Fig. 10 (B). Specifically in the spectrum, it is possible to verify two things, the equivalence of both current sources for the job to process the BIS analysis and the reduction in harmonic voltage strength with the frequency’s increment. This last effect is compatible with the capacitive part of Cole’s electrical model.

The higher ringing in Fig. 9 (A) and Fig. 9 (B) for SCMOSCS is due to its complex conjugated poles. The poles for SCMOSCS could be real or complex conjugated. Their values depend on the transistor dimensions and load. However, Fig. 10 (A) and Fig. 10 (B) shows that ringing does not cause an error in spectrum analysis. In addition, time domain BIS analysis is performed in a well-defined bandwidth, so the effect of these transitions is unlikely to affect the result.

Fig. 11 shows the results of the spectral analysis of the load impedance. The analysis shows that both sources produce results in line with the expected value (Nominal). However, the SCMOSCS has some advantages over the EHCS source when dealing with pseudo-random signals. Looking at Fig. 11 (A) it can be seen that the measured impedance is around 500 Ω at low frequencies and tends towards 250 Ω at high frequencies. These are the correct values, given that the current that causes the impedance between electrodes 3 and 4 is three times lower than the output current of the source, and that the impedance was calculated using the voltage between electrodes 3 and 4 and the output current of the sources. It is important to note that the frequencies to be considered in order to interpret Fig. 11 (A) and (B) are those corresponding to the peaks of the current or voltage spectrum shown in Fig. 10 and are marked by circles. In addition, the error about 3 degrees in load’s phase in comparison with the nominal value observed in Fig. 11 (B) is equal for both sources analysed.

## Conclusions

The Switching CMOS Current Source (SCMOSCS) is considered to be a superior alternative to the Enhanced Howland Current Source (EHCS) in time domain impedance spectroscopy for VLSI design. This is due to its numerous advantages that are particularly beneficial for battery-powered devices such as wearable devices. These advantages include low power consumption, low supply voltage, high output dynamic range, high output current accuracy, and high output impedance.

In addition to these features, the SCMOSCS also offers superior current precision, which eliminates the need for load current measurement hardware typically used in impedance spectroscopy. This makes it an excellent choice for wearable devices.Furthermore, when it comes to tetrapolar electrode impedance spectroscopy, the SCMOSCS represents a significant advancement over the pseudotetra-polar electrodes commonly used with the EHCS. The SCMOSCS is capable of effectively operating with tetra-polar electrodes, which are known for their accuracy in complex impedance measurements. This highlights the suitability of the SCMOSCS for more precise and reliable measurements in various applications, including medical diagnostics and material characterization.

However, it is worth noting that there is currently a lack of available design equations for the SCMOSCS in the existing literature. This presents an opportunity for future research to develop these equations. By doing so, not only would the design process be streamlined, but the scalability and applicability of the SCMOSCS in CMOS front-end designs for impedance spectroscopy would also be enhanced.
